# The Impact of *miR-21-5p*, *miR-145-5p* and *miR-382-5p* Expression in Gastric Adenocarcinoma Cells on Lymphatic Spread Capability

**DOI:** 10.3390/biomedicines13102393

**Published:** 2025-09-29

**Authors:** Maciej Ciesielski, Marzena Anna Lewandowska, Mariusz Szajewski, Krzysztof Pastuszak, Piotr Kurek, Jacek Zieliński, Jakub Walczak, Rafał Pęksa, Wiesław Janusz Kruszewski

**Affiliations:** 1 Department of Oncological Surgery, Gdynia Oncology Centre, 81-519 Gdynia, Poland; mariusz.szajewski@gumed.edu.pl (M.S.); piotr_kurek@gumed.edu.pl (P.K.); jwalczak@szpitalepomorskie.eu (J.W.); wieslaw.kruszewski@gumed.edu.pl (W.J.K.); 2 Department of Oncological Surgery, Faculty of Health Sciences with the Institute of Maritime and Tropical Medicine, Medical University of Gdańsk, 81-519 Gdynia, Poland; 3 Molecular Oncology and Genetics Department, Innovative Medical Forum, Łukaszczyk Oncology Centre, 85-796 Bydgoszcz, Poland; lewandowskam@co.bydgoszcz.pl; 4 Department of Thoracic Surgery and Tumors, Ludwik Rydygier Collegium Medicum in Bydgoszcz, Nicolaus Copernicus University, 85-067 Bydgoszcz, Poland; 5 Department of Algorithms and System Modelling, Gdańsk University of Technology, 80-233 Gdańsk, Poland; krzpastu@pg.edu.pl; 6 Laboratory of Translational Oncology, Intercollegiate Faculty of Biotechnology, University of Gdańsk and Medical University of Gdańsk, 80-307 Gdańsk, Poland; 7 Center of Biostatistics and Bioinformatics, Medical University of Gdańsk, 80-211 Gdańsk, Poland; 8 Department of General and Oncological Surgery, Janusz Korczak Specialist Hospital, 76-200 Słupsk, Poland; jaziel@gumed.edu.pl; 9 2nd Division of Radiology, Faculty of Health Sciences with the Institute of Maritime and Tropical Medicine, Medical University of Gdańsk, 80-210 Gdańsk, Poland; 10 Department of Pathomorphology, Faculty of Medicine, Medical University of Gdańsk, 80-210 Gdańsk, Poland; rafal.peksa@gumed.edu.pl

**Keywords:** microRNA, biomarkers, gastric adenocarcinoma, prognostic factors

## Abstract

**Objectives**: *miR-21-5p*, *miR-145-5p* and *miR-382-5p* have been associated with angiogenesis, which plays a central role in tumor growth and metastasis formation. The aim of the study was to determine whether expression of these three potentially angiogenic miRNAs is related to the lymphatic spread capability of gastric adenocarcinoma and patient survival. **Methods**: Pathoclinical data of 123 patients who underwent elective gastric resection for adenocarcinoma between 1 August 2006 and 31 December 2013 were retrospectively retrieved. The major concerns were the total number of lymph nodes retrieved, the number of positive nodes, depth of the tumor invasion to the stomach wall, pTNM stage of the disease, Lauren histological tumor type, presence of a mucinous component in the cancer tissue, tumor location in the stomach and survival outcome. The cancer tissues of patients were examined for the expression levels of *miR-21-5p*, *miR-145-5p* and *miR-382-5p*. **Results**: Elevated *hsa-miR-21-5p* expression levels and downregulated *hsa-miR-145-5p* levels were observed in patients with a higher pT stage, lymph node metastasis and advanced pTNM stage. Additionally, *hsa-miR-145-5p* expression was lower in patients with cardia involvement and a Lauren intestinal-type carcinoma. *hsa-miR-382-5p* levels were higher in patients with non-mucinous gastric carcinoma. Both *hsa-miR-145-5p* and *hsa-miR-21-5p* were predictors of the presence of node metastasis, even when adjusted for pT status. *hsa-miR-145-5p* was significantly associated with improved survival. *hsa-miR-145-5p* was significantly associated with an increased probability of surviving 3 years, while increased *hsa-miR-21* expression was significantly associated with reduced 3-year survival. All these associations were confirmed in multivariate models, which also included pT and M staging. **Conclusions**: The upregulation of *miR-21-5p* and downregulation of *miR-145-5p* are independent prognostic factors for lymph node metastasis and could serve as specific biomarkers of the lymphatic spread of gastric adenocarcinoma. *miR-145-5p* downregulation is an independent prognostic factor for overall survival.

## 1. Introduction

Despite the declining incidence and development of new treatment modalities, gastric cancer remains one of the deadliest oncological challenges [1]. Biological heterogeneity of the disease restricts prognostic predictions. One of the main causes of treatment failure is intense lymph node metastasis formation [2]. It is difficult to diagnose at an early stage, and the molecular pathways underlying lymphatic spread remain poorly understood [3]. Therefore, there is an increasing demand for novel biomarkers to predict the occurrence of lymph node metastases. Proper anticipation of lymphatic spread capability could increase the efficacy of comprehensive personalized patient management.

MicroRNAs are small non-coding nucleotides responsible for homeostatic regulation of gene expression and adaptation to transient changes in the cell microenvironment [4,5,6]. In pathological conditions, they play a remarkable role in tumorigenesis by altering the expression of oncogenes and tumor suppressors [5,6,7]. Specific miRNAs have been found to be related to tumor formation [4,8,9,10], angiogenesis [3,4,8,10,11,12], lymphatic spread [3], survival [4,13] and response to treatment [10,11,12,14]. Disorders of miRNA have been found in almost all types of cancer, and in every specific tumor, there is a distinct microRNA signature [4,8]. Two features make microRNAs unique: (i) stability in the tumor tissue and (ii) the possibility of miRNA modulation to reverse their oncogenic features [7]. Both are recognized as having substantial clinical potential as novel prognostic and predictive biomarkers in cancer diagnosis and treatment [4,8,9,10,15,16,17]. Their true diagnostic potential must be challenged in future clinical trials, and a combination of the most promising miRNAs as biomarkers with well-recognized pathoclinical parameters can enhance their value [6].

*miR-21-5p*, *miR-145-5p* and *miR-382-5p* have been associated with angiogenesis, which plays a central role in tumor growth and metastasis formation [7,10,11,12,18,19]. After reaching a diameter of about 1 mm, further expansion of the primary tumor is dependent on its own blood vessel formation [20]. For most carcinomas, including gastric cancer, lymphatic spread is preferential in comparison with blood vessel pathway dissemination, although lymphangiogenesis occurs after angiogenesis, and the formation of lymphatic spread is dependent on both angiogenetic and lymphangiogenetic factors [20].

The aim of the study was to determine whether expression of these three potentially angiogenic miRNAs is related to the lymphatic spread capability of gastric adenocarcinoma and patient survival in a population of Caucasian gastric cancer patients.

## 2. Materials and Methods

The analysis was designed to include all consecutive adult patients who underwent elective gastric resection for adenocarcinoma between 1 August 2006, and 31 December 2013, at the Oncological Surgery Department of the Medical University of Gdańsk. The exclusion criterion was the coexistence of any other malignancy, including lymphoma and stromal or neuroendocrine tumors.

The pathoclinical data of the patients were retrospectively retrieved, and all samples were the subject of a thorough histopathological reexamination at the Department of Pathomorphology, Medical University of Gdańsk. The major concerns were the total number of lymph nodes retrieved, the number of positive nodes, depth of the tumor invasion to the stomach wall, pTNM stage of the disease according to the 8th edition of the AJCC Cancer Staging Manual [21], Lauren histological tumor type, presence of mucinous component in the cancer tissue, tumor location in the stomach and survival outcome.

MicroRNA assays were performed at the Molecular Oncology and Genetics Department, IFM, Łukaszczyk Oncology Centre in Bydgoszcz.

The study was approved by the Independent Ethics Committee at the Medical University of Gdańsk (NKBBN/90/2017).

Among 144 archival primary tumor specimens, 20 cases were excluded because of insufficient tumor material (19 cases) or concomitant gastric lymphoma (1 case). Because of the failure of a miRNA assessment in one of the specimens, the final studied material consisted of 123 cases.

For statistical reasons, we combined patients with pTis-2 stages into one group and pT3-4 into the second group. Similarly, we combined TNM stages I and II into one group and III and IV into the second group.

Mortality data were acquired from the Polish Ministry of Digitalization on 1 November 2023.

Isolation of microRNA derived from 32 gastric adenocarcinoma patients (three groups: pT1-T2N1, pT3-T4N0 and pT3-T4N3) was performed using the miRCURY RNA isolation kit dedicated for FFPE samples (Exiqon, Qiagen, Copenhagen, Denmark), and quality and quantity control were performed (Bioanalizator, Agilent, Santa Clara, CA, USA). Some of the FFPE samples derived between 2007 and 2013 yield degraded RNA, which could affect NGS and qPCR accuracy; therefore, not all 32 samples were taken for NGS analysis. Firstly, miRNA quality was evaluated, and we chose 20 of 32 FFPE samples based on spectrophotometry. The final decision on which samples should be selected for NGS was based on a miRNA quality analysis performed using an Agilent 2100 Bioanalyzer. Therefore, 10 of 32 of the samples with the highest microRNA abundance and the best miRNA quality, isolated from the FFPE samples derived between 2007 and 2013 and representing all three groups, were chosen for microRNA next-generation sequencing profiling. RIN analysis was performed in service.

A total of 100 ng of total RNA was converted into microRNA NGS libraries (Exiqon Services, Qiagen, Copenhagen, Denmark). Adapters were ligated to the RNA, and then the RNA was converted to cDNA. The cDNA was amplified using PCR (18 cycles), during which the PCR indices were added. After the PCR, the samples were purified. Library preparation QC was performed using the Bioanalyzer 2100 (Agilent). Based on the quality of the inserts and the concentration measurements, the libraries were pooled in equimolar ratios. The pool was then size-selected using the LabChip XT (PerkinElmer, Singapore), aiming to select the fraction with the size corresponding to the microRNA libraries (~145 nt). The library pool(s) were quantified using the qPCR KAPA Library Quantification Kit (KAPA Biosystems, Wilmington, MA, USA). The library pool was then sequenced on a NextSeq 500 (Illumina, San Diego, CA, USA) sequencing instrument according to the manufacturer’s instructions.

For each sample, an average of 10 microRNA reads was obtained using 51-nucleotide single-end sequencing. Following sequencing, intensity correction, base calling and assigning of Q-scores was performed. Subsequently, data quality was checked. All 10 samples chosen for NGS sequencing showed overall high quality, with the vast majority of the data obtained presenting a Q-score of more than Q30. The average reads Q-scores and base Q-scores of the NGS sequencing data for the samples are presented in Supplementary Figure S1 and Figure S2, respectively. After sequencing, the adapters were trimmed off as part of the base calling. Trimming of the adapters from the dataset revealed one distinct peak representing microRNA (~18–23 nt). On the other hand, as expected, sequencing of old archival material showed additional reads of other lengths, possibly reflecting degradation products from other RNA species. Comparison of experimental groups was performed.

Using NormFinder (MOMA, Aarhus, Denmark) analysis, the most stably expressed microRNA in the gastric adenocarcinoma FFPE samples based on microRNA NGS analysis was established. We chose *hsa-miR-30c-5p*, *hsa-miR-125b-5p* and *has-miR125a-5p* based on the NGS experimental approach and *U6*, *UniSP3* and *UniSp6* as control microRNAs for qPCR normalization. For the selected microRNA validation from the NGS data, we chose microRNAs based on more than 2-fold up or downregulation, together with supporting evidence in the literature. A total of 11 microRNAs were selected for validation using qPCR NGS on a miRCURY LNA miRNA custom PCR panel with exiLENT SYBR Green Master Mix kit (Exiqon): *hsa-miR-375*, *hsa-miR-145-5p*, *hsa-miR-21-5p*, *hsa-miR-187-3p*, *hsa-miR-196a-5p*, *hsa-miR-708-3p*, *hsa-miR-142-3p*, *hsa-miR-27b-5p*, *hsa-miR-552-3p*, *hsa-miR-382-5p* and *hsa-miR-128-3p*. MicroRNA expression was assessed in 124 samples due to assessment failure in one specimen; the final studied material consisted of 123 cases. For the purpose of this study, we chose to describe the role of three miRNAs only, which, according to the literature, have been found to be related to angiogenesis: *hsa-miR-21-5p*, *hsa-miR-145-5p* and *hsa-miR-382-5p*.

Statistical analysis was conducted in R (version 4.3.2). Fisher’s test was used to assess binary variables. The Freeman–Halton extension was used for categorical variables with multiple levels. For quantitative variables, the normality of distribution was evaluated using the Shapiro–Wilk test. Since the miRNA expression data did not follow a normal distribution, the Wilcoxon test was used for pairwise comparisons. For comparisons with multiple groups, the Kruskal–Wallis test was used. The Schoenfeld test was used to assess the proportionality of hazards; the proportional hazards assumption was met for all covariates in both univariate and multivariate Cox regression models (*p* > 0.05 for hsa-miR-21-5p [univariate: 0.2373; multivariate: 0.1871], hsa-miR-145-5p [univariate: 0.5245; multivariate: 0.5819] and hsa-miR-382-5p [univariate: 0.7804; multivariate: 0.8543]). Hazard ratios were evaluated using Cox regression models. Logistic regression models were built to assess odds ratios. Correlations were assessed using the Pearson method if the data followed a normal distribution, and the Spearman method if otherwise. No data imputation was used. *p* < 0.05 was considered significant.

## 3. Results

### 3.1. Characteristics of the Study Subjects

The mean (median) age of patients in the studied group was 62.7 (63) years. There were 87 (70.7%) males and 36 (29.3%) females. All 123 patients underwent major surgical resection for gastric adenocarcinoma; there were a total of 104 (84.6%) gastrectomies and 19 (15.4%) subtotal resections. The extent of lymphadenectomy reported by the operating surgeon was D2 in 21 (17.1%) and D1 or D1+ in 122 (82.9%) patients. The cardia was involved in 42 (34.1%) cases, and the remaining tumors were located distally in the stomach. Data from histopathological reports and survival are collected in Table 1.

### 3.2. Univariate Analysis of the Correlation Between miRNA and Pathological Characteristics

Elevated *hsa-miR-21-5p* expression levels and downregulated *hsa-miR-145-5p* levels were observed in patients with a higher pT stage, lymph node metastasis and more advanced pTNM disease stage. Additionally, *hsa-miR-145-5p* expression was lower in patients with cardia involvement and Lauren intestinal-type carcinoma. A weak negative correlation was observed between *has-miR-145-5p* expression level and the number of nodes with metastasis. *hsa-miR-382-5p* levels were higher in patients with non-mucinous gastric carcinoma. Additionally, *hsa-miR-382-5p* expression was weakly positively correlated with the number of nodes with metastasis. No correlations were found between node ratio (ratio between the positive nodes and the total number of nodes resected) and any of the considered miRNAs. The statistics are presented in Table 2 and Table 3.

### 3.3. Multivariate Analysis of the Correlation Between miRNA and Pathological Characteristics

We further investigated the associations between miRNA expression levels and lymph node metastasis using logistic regression analyses. In univariate models, a doubling in *hsa-miR-145-5p* expression was associated with reduced odds of node metastasis presence (OR 0.572, 95% CI: 0.36–0.86, *p* = 0.0099), whereas a doubling in *hsa-miR-21-5p* expression was linked to increased odds (OR 1.646, 95% CI: 1.10–2.53, *p* = 0.0178). These findings persisted in multivariate models adjusted for pT status (*hsa-miR-145-5p*: adjusted OR 0.597, 95% CI: 0.38–0.89, *p* = 0.0174; *hsa-miR-21-5p*: adjusted OR 1.613, 95% CI: 1.07–2.49, *p* = 0.025). The relationship between miRNA expression and the number of node metastases was also evaluated. Neither *hsa-miR-145-5p* nor *hsa-miR-21-5p* significantly predicted the metastasis count. However, a doubling of *hsa-miR-382-5p* expression was significantly associated with a doubling of the number of node metastases in multivariate models adjusted for pT status (*p* = 0.0351; results not tabulated). Sex did not emerge as a significant covariate in any models, while age was a significant prognostic factor in several; however, inclusion of age and sex resulted in only minor changes to the miRNA effect estimates, with no impact on their statistical significance. Statistics for the presence of node metastasis (adjusted for tumor characteristics) are presented in Table 4. Detailed results from these additional analyses (further adjusted for age and sex) are provided in Table 5.

### 3.4. Analysis of the Correlation Between miRNA and Survival

We then analyzed the impact of selected miRNA expression levels on overall survival using univariate Cox proportional hazards regression models for each miRNA. Notably, higher hsa-miR-145-5p expression was significantly associated with improved survival, with a hazard ratio (HR) of 0.78 (95% CI: 0.64–0.95, *p* = 0.013) per doubling of expression levels. This association persisted in multivariate Cox models adjusted for pT and M staging (adjusted HR: 0.79, 95% CI: 0.65–0.97, *p* = 0.025).

**Figure 1:**
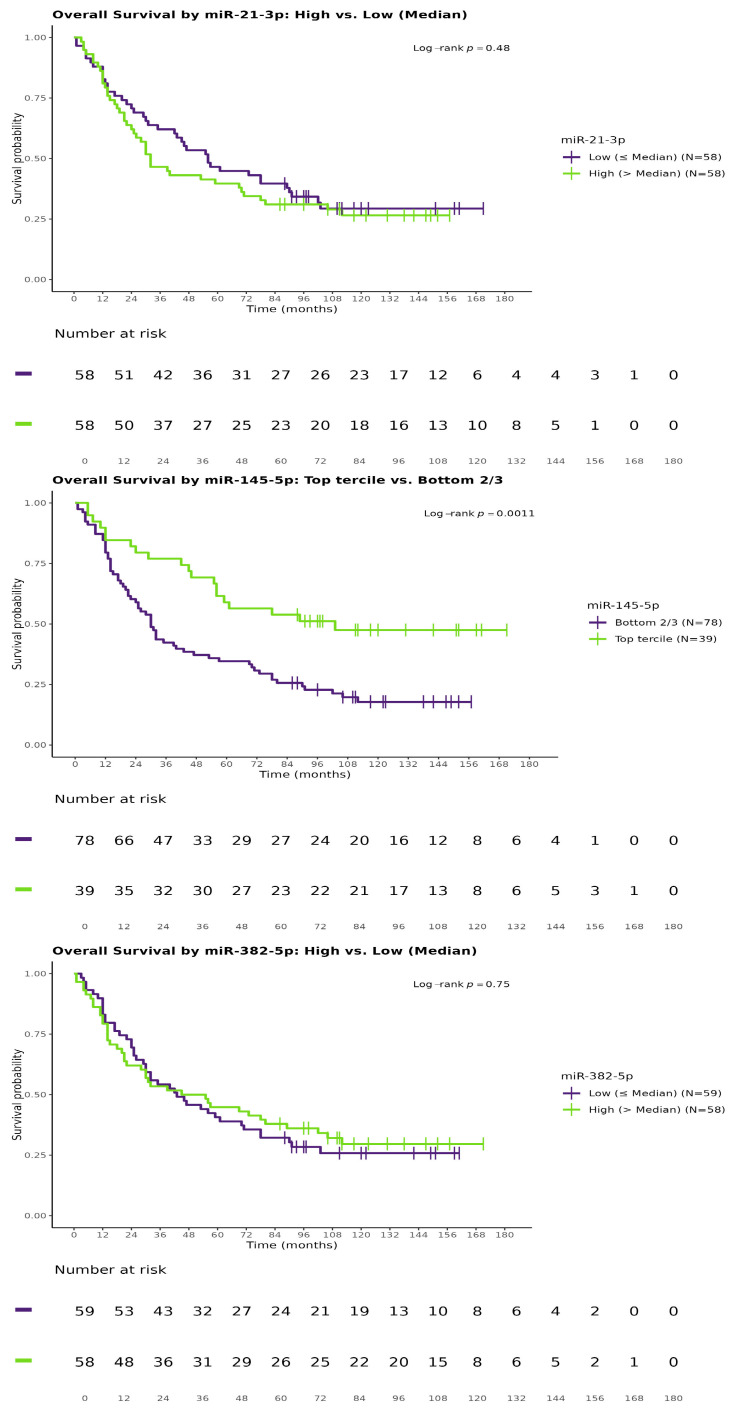
Kaplan–Meier curves for survival probability.

We next evaluated the influence of miRNA expression on the probability of surviving specific time points (1, 2, 3, 4, and 5 years) using logistic regression. No miRNAs showed significant associations with 1-year (all *p* > 0.1) or 2-year survival (hsa-miR-145-5p: OR 1.441, 95% CI: 1.00–2.12, *p* = 0.0546 per doubling; results not tabulated). However, higher hsa-miR-145-5p expression was linked to an increased likelihood of 3-year survival (OR 1.863, 95% CI: 1.28–2.82, *p* = 0.0019), while elevated hsa-miR-21-5p expression was associated with reduced 3-year survival (OR 0.651, 95% CI: 0.45–0.93, *p* = 0.0219). These findings were robust in multivariate models adjusted for pT and M staging. For 4- and 5-year survival, hsa-miR-145-5p was significant in univariate analyses (4-year: OR 1.738, 95% CI: 1.20–2.61, *p* = 0.005; 5-year: OR 1.521, 95% CI: 1.06–2.25, *p* = 0.0273), but only the 4-year association was confirmed in multivariate models (adjusted OR 1.700, 95% CI: 1.16–2.58, *p* = 0.0087; 5-year adjusted OR 1.459, 95% CI: 1.01–2.18, *p* = 0.0539, approaching significance). hsa-miR-382-5p expression showed no significant associations with any survival outcomes. Detailed statistics are presented in Table 4 (adjusted for tumor characteristics) and Table 5 (additionally adjusted for age and sex). Kaplan–Meier survival curves are shown in Figure 1.

## 4. Discussion

Recently, the Nobel Prize was awarded (jointly to Victor Ambros and Gary Ruvkun) for the discovery of microRNA and its role in post-transcriptional gene regulation, but a detailed understanding of the role of microRNA in carcinogenesis is still ongoing [22]. The influence of *miR-21* on gastric cancer progression has been well documented [6,9]. Simultaneous suppression of *miR-21-5p* combined with *miR-145-5p* overexpression in vitro has been shown to decrease the proliferation of gastric cancer cells more efficiently than *miR-21-5p* suppression alone [23]. It was shown in vivo and in vitro that *miR-145-5p* is downregulated in gastric cancer and that it inhibits gastric cancer formation [10,17]. Our findings showed that the upregulation of *miR-21-5p* and downregulation of *miR-145-5p* in primary tumor cells have a statistically significant association with lymph nodes metastasis formation, independent of the depth of the primary tumor invasion into the stomach wall (pT stage). The impact of *miR-382-5p* expression on the presence of lymph node metastasis did not reach the level of statistical significance (*p* = 0.09); however, upregulation of *miR-382-5p* was statistically correlated with the number of positive nodes (Table 3). Multiple studies have demonstrated an association between numerous miRNAs and lymphatic status in univariate analyses [24,25,26,27]. It should be noted that higher pT status in gastric cancer increases the risk of lymph node involvement as a natural consequence of cancer progression, and up- or downregulation of these miRNAs might be associated with—or occur in the environment of—the more advanced tumor [28]. We also found an association between *miR-21-5p* upregulation and *miR-145-5p* downregulation and poor survival. For *miR-21-5p*, statistical calculations showed significant associations with reduced 3-year survival, both in the univariate and multivariate models, while for *miR-145-5p*, its downregulation had a significant impact on worse 3-year, 4-year, 5-year and overall survival. All associations were confirmed in the multivariate models, except for 5-year survival.

*miR-21* belongs to the best-characterized cancer-associated microRNA, and the oncogenic pathways activated by *miR-21* have already been quite widely described [4,5,6,8,9,14,23]. *miR-21* is known to be associated with the regulation of multiple tumorigenic gene expressions, and it was found to be upregulated in nearly all solid tumors, including gastric cancer [3,14,16,23,29]. Its downregulation in saliva may indicate gastric cancer occurrence [15]. Estimated *miR-21-5p* levels in extracellular vesicles from the blood serum of gastric cancer patients may serve as a promising predictive and prognostic factor [16]. *miR-21-5p* is significantly elevated in the serum of cancer patients before surgery compared with its expression in healthy volunteers and significantly decreased after surgery [16]. In one study, it was overexpressed in 92% of gastric cancer cases [30]. The level of *miR-21* expression is significantly related to the advanced stage of the disease [3]; however, it is also frequently overexpressed in Helicobacter-pylori-infected mucosa [14], suggesting the occurrence of miRNA dysregulation at an early stage of the disease. An in vitro study showed that *miR-21* had been induced by Helicobacter pylori infection, and knockdown of *miR-21* had remarkably decreased gastric cell invasion and migration and increased apoptosis [14]. Therefore, *miR-21* could have multiple functions in both cancer genesis and progression [5].

To our knowledge, only one other study has, to date, reported that high expression of *miR-21* is an independent prognostic factor for lymphatic spread capability [31]. In the study conducted on 86 gastric adenocarcinoma specimens, it was found in the multivariate analysis that high expression of *miR-21* was related to lymph node metastasis and a higher pTNM score [31].

A meta-analysis of eight studies concerning the prognostic value of *miR-21* found an association between high *miR-21* expression and poor tumor differentiation, lymph node metastasis, higher TNM stage and poor survival [25]. The authors, however, did not carry out a multivariate analysis to conclude whether *miR-21* could work as an independent prognostic factor for lymphatic spread [25]. An original study on 50 gastric cancer specimens found an association between high expression of *miR-21* and metastases, as well as overall survival, but also between high *miR-21* expression and tumor size [26]. In another meta-analysis concerning, among others, *miR-21* and *miR-145*, a high expression of *miR-21* and low expression of *miR-145* were associated with shorter overall survival [13].

In contrast to *miR-21*, *miR-145* is usually downregulated in gastric cancer tissue, and its overexpression suppresses tumorigenesis [10,12,17]. In a study involving 145 patients, low expression of *miR-145-5p* was significantly associated with both lymph node and distant metastasis, as well as overall survival. In their multivariate analysis, *miR-145-5p* was an independent prognostic factor for overall survival only [27].

The results of another study suggest that *miR-145-5p* targets *SERPINE1* and inhibits gastric cancer development [17]. It was also shown that *miR-145-5p* affects gastric cancer progression via the ANGPT2/NLR axis and participates in angiogenesis, proliferation and migration of cancer cells [12]. In another study, *miR-145* was found to increase the sensitivity of gastric adenocarcinoma cell line MKN-1 to 5-FU, which remains one of the main chemotherapeutic agents applied in gastric cancer [32].

What seems to be very interesting about this particular tumor-suppressing miRNA is that it was found to be overexpressed in stromal fibroblasts with a scirrhous-type histology of gastric adenocarcinoma, which is well recognized to be associated with extremely poor patient prognosis [33]. The explanation given by the authors is that it participates in the transforming growth factor-β pathway by enhancing the expression of α-smooth muscle actin, which eventually results in the activation of peri-tumoral fibroblasts and cancer progression [33]. In our study, apart from the impact on lymphatic spread, we found an association between *miR-145-5p* downregulation and cardia involvement and intestinal type according to the Lauren classification.

In contrast to *miR-21* and *miR-145*, *miR-382* is not well described in the literature. It belongs to non-coding RNAs that induce ferroptosis, a type of non-apoptotic cell death [34]. It was chosen for this study because it has been known to promote angiogenesis [19]. In an experimental study on MKN-1 human gastric cancer cell lines, the authors found that *miR-382* upregulation had been induced by hypoxia, and it had acted like an angiogenic oncogene by repressing the well-recognized tumor suppressor, the phosphatase and tensin homolog (PTEN) gene [19]. Hypoxia is known to increase angiogenesis, limit the efficacy of oncologic treatment and the adaptation of malignant cells to hypoxic conditions, all of which result in an increase in tumor aggressiveness [35]. The same authors working on the clinical material of gastric adenocarcinoma found a correlation between the expression of *miR-382* and higher T-stage, N-stage, pTNM stage, lymphovascular, venous and perineural invasion, as well as overall survival [35]. However, the results of their study were not subjected to multivariate analysis, and the real impact of *miR-382* upregulation on gastric cancer progression and prognosis in their material remained unknown [35]. In our study, upregulation of *miR-382* was statistically related only to the number of metastatic nodes, and we failed to demonstrate the impact of *miR-382* on the presence of lymph node metastasis in both univariate and multivariate models. However, downregulation of *miR-382* was associated with the mucinous component of gastric tumors.

The results of another study concerning the impact of different microRNAs on lymph node involvement in gastric cancer also lacked a multivariate analysis [24]. The authors examined the impact of six miRNA expressions on predicting lymph node metastasis in 102 gastric tumor samples [24]. They identified four miRNAs (*miR-27b*, *miR-128*, *miR-100* and *miR-214*) that were associated with the presence of positive nodes [24]. Some of the studied miRNAs were also associated with pT stage [24]. The authors did not conclude whether these four miRNAs could be recognized as an independent prognostic factor for the occurrence of lymph node metastases [24].

In conclusion, it is noting that the vast majority of studies concerning the value of miRNA expression in gastric cancer cells as a prognostic and predictive biomarker, including all the studies citied in this paper, come from Asia [8,11,12,13,14,15,16,17,23,24,25,26,27,29,30,31,32,33,34,35]. Gastric cancer is a highly heterogeneous disease, and many disparities exist concerning gastric adenocarcinoma incidence, risk factors, histology and molecular characteristics between different ethnic groups and races [36]. Our study, to our knowledge, is the first performed on a Caucasian cohort to reveal the impact of *miR-21-5p* and *miR-145-5p* expression on lymphatic spread capability.

### Limitations of the Study

There are some limitations to our study. First, because of its retrospective nature, we were not able to gather fair data concerning neoadjuvant and adjuvant therapy. Second, we did not assess miRNA expression in the adjacent non-tumor tissue mucosa for a reference to the normal expression level. We also did not collect tumor types according to the Borrmann classification. Last, another limitation of our study was the lack of a separate patient population for NGS analysis and qPCR validation and the use of archival FFPE material of gastric resections performed between 2007 and 2013, which could have an impact on miRNA degradation and miRNA selection. In addition, at the time when the miRNA NGS experiment was performed (March 2017), a typical microRNA sequencing experiment yielded approximately 10–60% microRNAs mapping to the reference genome, which, together with archival FFPE miRNAs, limited the selection of miRNAs.

## 5. Conclusions

The upregulation of *miR-21-5p* and downregulation of *miR-145-5p* are independent prognostic factors for lymph node metastasis and could serve as specific biomarkers of lymphatic spread of gastric adenocarcinoma. *miR-145-5p* downregulation is an independent prognostic factor for overall survival.

## Figures and Tables

**Table 1:** Histopathological characteristics and survival in the studied group.

**Histopathological Feature/Survival**	**n = 123**
pTNM	
I	22 (17.9%)
II	34 (27.6%)
III	62 (50.4%)
IV	5 (4.1%)
N+	88 (71.6%)
Mean (median) number of metastatic lymph nodes	6.0 (3)
Mean (median) number of resected lymph nodes	21.8 (20)
Mucinous component	28 (22.8%)
Lauren histopathological type	
1 Intestinal	60 (48.8%)
2 Diffuse	35 (28.5%)
3 Mixed	28 (22.8%)
Survival	
12 months	101 (82.1%)
24 months	79 (64.2%)
36 months	63 (51.2%)
48 months	56 (45.5%)
60 months	50 (40.7%)

**Table 2:** miRNA expression levels depending on the clinical and histopathological features.

		***hsa-miR-21-5p***	***hsa-miR-145-5p***	***hsa-miR-382-5p***
pT	is-2 [n = 33]	2.57 (0.2–16.23)	22.34 (3.26–229.1)	0.01 (0–0.21)
	3–4 [n = 90]	7.72 (0.89–26.3)	8.68 (0.91–26.4)	0.02 (0–0.66)
*p*		<0.0001	<0.0001	0.0984
pN	N0 [n = 35]	3.28 (0.89–26.2)	17.17 (1.67–229.1)	0.01 (0–0.21)
	N+ [n = 88]	7.25 (0.2–26.3)	10.58 (0.91–29.87)	0.02 (0–0.66)
*p*		0.0097	0.0077	0.0948
M	M0 [n = 118]	6.49 (0.2–26.3)	12.05 (0.91–229.1)	0.02 (0–0.66)
	M1 [n = 5]	7.32 (2.04–12.66)	11.92 (5.25–22.78)	0.02 (0–0.41)
*p*		0.9897	0.8376	0.9591
pTNM stage	I–II [n = 56]	4.78 (0.2–26.3)	13.54 (1.67–229.1)	0.01 (0–0.21)
	III–IV [n = 67]	7.71 (1.58–24.71)	9.66 (0.91–26.4)	0.02 (0–0.66)
*p*		0.0072	0.0211	0.0718
Mucinous	No [n = 95]	6.72 (0.2–26.3)	10.19 (0.91–229.1)	0.02 (0–0.66)
	Yes [n = 28]	5.91 (1.24–17.88)	14.76 (2.51–39.65)	0.01 (0–0.07)
*p*		0.9781	0.0563	0.0262
Tumor location	Cardia [n = 42]	7.26 (1.06–26.3)	8.19 (0.91–29.92)	0.01 (0–0.49)
	Other [n = 81]	5.63 (0.2–26.2)	13.14 (1.28–229.1)	0.02 (0–0.66)
*p*		0.0646	0.0055	0.2395
Lauren	Intestinal [n = 59]	7.1 (0.2–26.3)	8.31 (0.91–229.1)	0.01 (0–0.21)
	Diffuse [n = 35]	4.75 (1.24–19.42)	14.8 (4.7–39.65)	0.02 (0–0.66)
	Mixed [n = 27]	4.25 (1.68–24.71)	11.71 (2.37–27.24)	0.03 (0–0.49)
*p*		0.1814	0.0037	0.1607

**Table 3:** Correlation between miRNA expression levels and number of positive nodes.

	***hsa-miR-21-5p***		***hsa-miR-145-5p***		***hsa-miR-382-5p***	
	**r**	***p***	**r**	***p***	**r**	***p***
Number of nodes with metastasis	0.15	0.0992	−0.18	0.046	0.2	0.0262

**Table 4:** Univariate and multivariate hazard/odds ratios for miRNA expression levels as predictors of survival and presence of node metastasis in gastric adenocarcinoma (multivariate models adjusted for tumor characteristics: pT status for node metastasis; pT and M staging for survival outcomes).

**miRNA**	**Outcome**	**HR/OR**	**95% CI**	***p*** **-Value**	**Adjusted HR/OR**	**95% CI Adjusted**	**Adjusted *p*-Value**
*hsa-miR-145-5p*	Hazard ratio	0.78	0.64–0.95	0.013	0.79	0.65–0.97	0.025
	3-year survival	1.863	1.28–2.82	0.0019	1.851	1.26–2.83	0.0028
	4-year survival	1.738	1.2–2.61	0.005	1.7	1.16–2.58	0.0087
	5-year survival	1.521	1.06–2.25	0.0273	1.459	1.01–2.18	0.0539
	Node metastasis	0.572	0.36–0.86	0.0099	0.597	0.38–0.89	0.0174
*hsa-miR-21-5p*	Hazard ratio	1.12	0.91–1.39	0.2746	1.1	0.88–1.37	0.4022
	3-year survival	0.651	0.45–0.93	0.0219	0.657	0.45–0.95	0.0279
	4-year survival	0.739	0.51–1.05	0.0989	0.754	0.52–1.09	0.1332
	5-year survival	0.817	0.57–1.17	0.2711	0.845	0.58–1.23	0.3761
	Node metastasis	1.646	1.1–2.53	0.0178	1.613	1.07–2.49	0.025
*hsa-miR-382-5p*	Hazard ratio	0.6	0.09–4.07	0.602	0.43	0.06–2.98	0.3932
	3-year survival	0.575	0.02–12.06	0.7176	0.898	0.03–21.34	0.9467
	4-year survival	1.271	0.06–27.73	0.8751	2.25	0.08–56.4	0.6146
	5-year survival	2.045	0.09–45.71	0.6404	4.434	-	0.3696
	Node metastasis	49.627	-	0.1475	49.658	-	0.1527

**Table 5:** miRNA levels as predictors of survival and nodal involvement in gastric adenocarcinoma: univariate and multivariate hazard/odds ratios (adjusted for patient age, sex, pT status for node metastasis; pT and M staging for survival outcomes).

**miRNA**	**Outcome**	**HR/OR**	**95% CI**	***p*** **-Value**	**Adjusted HR/OR**	**95% CI Adjusted**	**Adjusted *p*-Value**
*hsa-miR-145-5p*	Hazard ratio	0.78	0.64–0.95	0.013	0.77	0.62–0.96	0.0222
	3-year survival	1.863	1.28–2.82	0.0019	1.871	1.25–2.91	0.0034
	4-year survival	1.738	1.2–2.61	0.005	1.705	1.15–2.62	0.0103
	5-year survival	1.521	1.06–2.25	0.0273	1.463	1.00–2.21	0.0576
	Node metastasis	0.572	0.36–0.86	0.0099	0.544	0.33–0.84	0.0093
*hsa-miR-21-5p*	Hazard ratio	1.12	0.91–1.39	0.2746	1.06	0.84–1.33	0.6279
	3-year survival	0.651	0.45–0.93	0.0219	0.698	0.47–1.02	0.0674
	4-year survival	0.739	0.51–1.05	0.0989	0.804	0.55–1.18	0.2636
	5-year survival	0.817	0.57–1.17	0.2711	0.906	0.61–1.34	0.62
	Node metastasis	1.646	1.1–2.53	0.0178	1.756	1.15–2.76	0.0112
*hsa-miR-382-5p*	Hazard ratio	0.6	0.09–4.07	0.602	0.49	0.07–3.23	0.4593
	3-year survival	0.575	0.02–12.06	0.7176	0.737	0.02–19.64	0.8584
	4-year survival	1.271	0.06–27.73	0.8751	1.932	0.06–53.55	0.6988
	5-year survival	2.045	0.09–45.71	0.6404	3.693	-	0.458
	Node metastasis	49.627	-	0.1475	40.766	-	0.1738

## Data Availability

The original contributions presented in this study are included in the article. Further inquiries can be directed to the corresponding author.

## Supplementary Materials

The following supporting information can be downloaded at: https://www.mdpi.com/article/10.3390/biomedicines13102393/s1; Figure S1: The average reads Q-scores of the NGS sequencing data for the samples; Figure S2: The average base Q-scores of the NGS sequencing data for the samples.

## Abbreviations

The following abbreviations are used in this manuscript:

FFPE	Formalin-fixed paraffin-embedded
NGS	Next-generation sequencing
qPCR	Quantitative polymerase chain reaction
OR	Odds ratio
HR	Hazard ratio
AJCC	American Joint Committee on Cancer
pTNM	Pathological tumor-node-metastasis
MOMA	Aarhus University Molecular Medicine
5-FU	5-Fluorouracil
PTEN	Phosphatase and tensin homolog
ANGPT2	Angiopoietin-2
NLR	Nod-like receptor
