# Supplementary figures and images for: The Impact of miR-21-5p, miR-145-5p and miR-382-5p Expression in Gastric Adenocarcinoma Cells on Lymphatic Spread Capability
Source: Biomedicines. 2025 Sep 29;13(10):2393. doi: 10.3390/biomedicines13102393 (PMC12561494; doi:10.3390/biomedicines13102393)

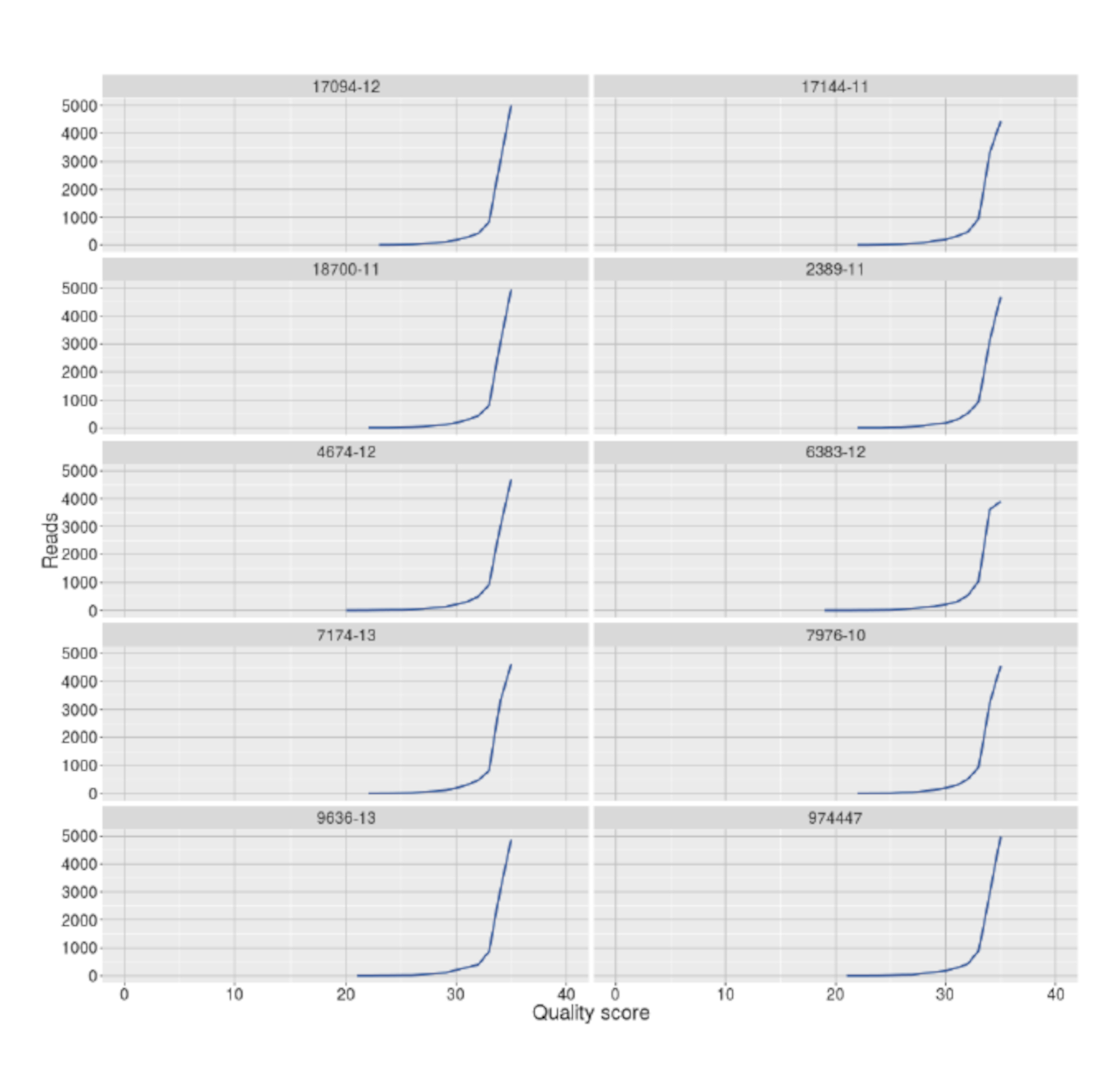

Supplement: Supplementary file 1 [file biomedicines-13-02393-s001.zip › Supplementary Figure S1.jpeg]

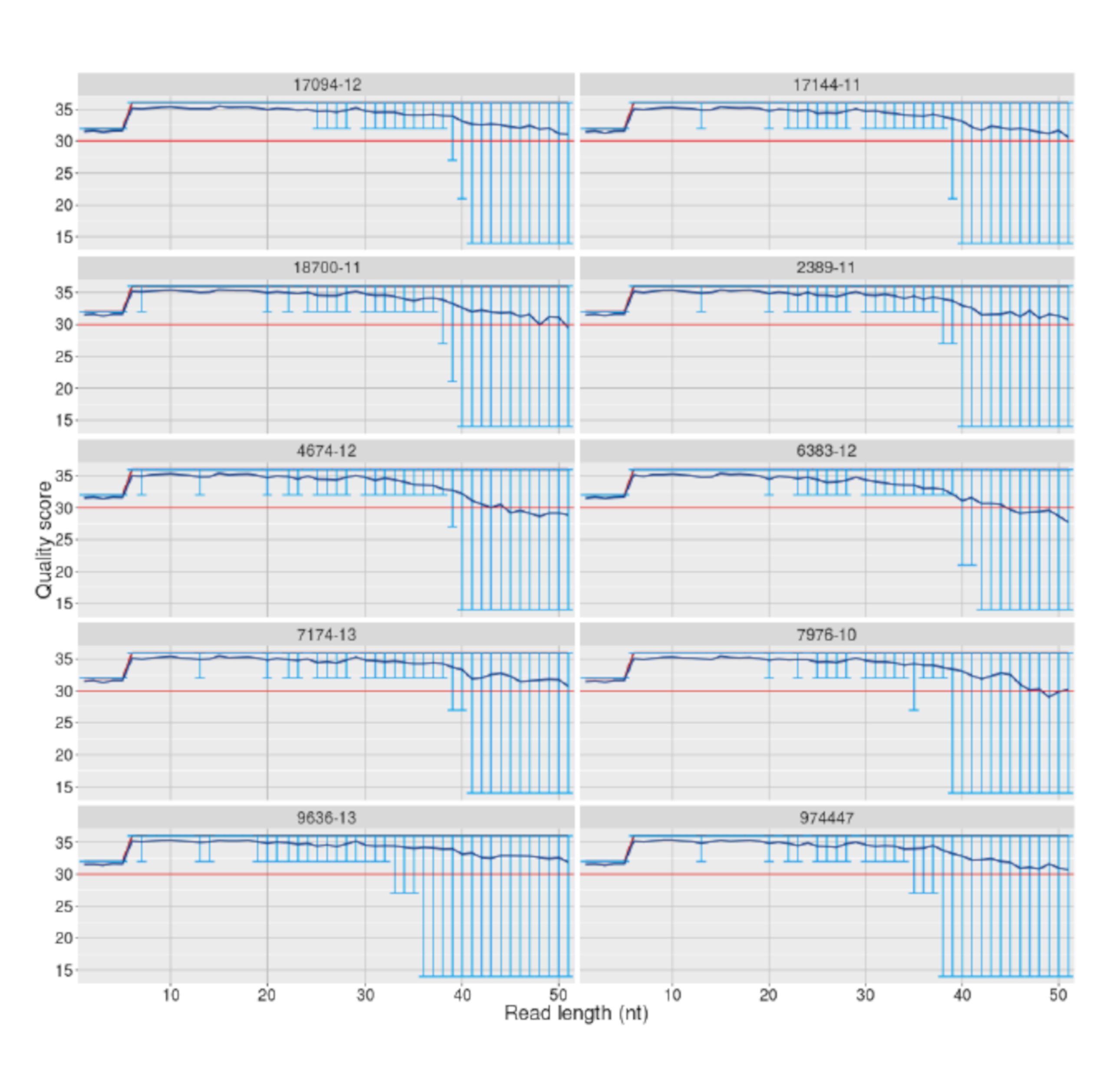

Supplement: Supplementary file 1 [file biomedicines-13-02393-s001.zip › Supplementary Figure S2.jpeg]
